# A special RELationship between sugar and tumor-infiltrating regulatory T cells

**DOI:** 10.1038/s41423-024-01248-5

**Published:** 2024-12-17

**Authors:** Ingo Schmitz

**Affiliations:** Department of Molecular Immunology, Ruhr University Bochum, Bochum, Germany

**Keywords:** NF-kappaB, Regulatory T cells

In this context of *Cellular and Molecular Immunology*, Sharma and coworkers reported that the glucose transporter Glut3 is specifically expressed in tumor-infiltrating (TIL) regulatory T (Treg) cells [1]. The upregulation of Glut3 expression in TIL-Treg cells increased their glucose uptake, which was then used to modify the activity of signaling proteins. The authors specifically investigated the NF-κB subunit c-Rel and showed that its posttranslational modification with *O*-GlcNAc contributes to the immunosuppressive environment of tumors, opening up possibilities for novel therapeutic approaches.

In the past two decades, substantial progress has been made in the field of immunometabolism. Early on, it was realized that naïve T cells switch their metabolism from oxidative phosphorylation to aerobic glycolysis during activation [2], which is akin to the Warburg effect in cancer cells [3]. Although glycolysis produces less ATP than does oxidative phosphorylation, the metabolic switch releases metabolites as building blocks for the synthesis of novel macromolecules or for posttranslational modifications (PTMs) of proteins. These metabolite-based PTMs regulate the activity of signaling mediators, including acylation, succinylation, and *O*-GlcNAcylation [4]. Hence, signal transduction (e.g., via the TCR during T-cell activation) influences metabolism, and metabolism, in turn, influences signaling.

*O*-GlcNAcylation is a reversible PTM that involves the modification of serine and threonine residues by *O*-linked *N*-acetylglucosamine (*O*-GlcNAc) [4]. It is mediated by *O*-GlcNAc transferase (OGT), which uses UDP-GlcNAc as a substrate for the modification of target proteins. The removal of O-GlcNAc is catalyzed by the *O*-GlcNACase (OGA) enzyme. *O*-GlcNAcylation regulates processes such as insulin signaling, transcription, and epigenetic changes [4]. During lymphocyte activation, the activities of the transcription factors NF-κB and NFATc1 are regulated by *O*-GlcNAcylation [5].

In addition to T-cell activation, changes in T-cell metabolism are crucial for T-cell differentiation and effector functions. For example, the glycolytic enzyme GAPDH functions as an RNA-binding protein crucial for the production of IFNγ, which is a key cytokine of Th1 effector cells [6]. Importantly, the Warburg-like metabolic switch upon T-cell activation restricts GAPDH to its glycolytic function and prevents its RNA-binding activity, allowing IFNγ to be expressed. This metabolic switch is also facilitated by the transcriptional induction of glucose transporter (Glut) gene expression. T-cell receptor signaling leads to the activation of the transcription factors c-Myc and HIF-1α, which activate a gene expression program that induces aerobic glycolysis [7, 8]. Among the induced genes is Glut1, which has been shown to be crucial for the upregulation of glycolysis, T-cell activation, and effector functions of inflammatory T-cell subsets [7–9]. In addition to Glut1, activated lymphocytes can express Glut3, which is otherwise expressed in the nervous system [9, 10]. Glut3 is highly expressed in Th17 cells and crucial for the effector functions of these cells [11]. Glut3-mediated glucose uptake allows mitochondrial glucose oxidation and acetyl-CoA production, which are needed for epigenetic changes that regulate the expression of proinflammatory genes. Therefore, the inhibition of Glut3 expression or ActylCoA production prevents autoimmunity [11]. Thus, Glut3-dependent acetyl-CoA generation is defined as a metabolic checkpoint. In addition, overexpression of Glut3 in CD8+ cytotoxic T cells enhances their effector function and antitumor activity by increasing glucose uptake and energy storage in glycogen deposits [12].

A study by Sharma et al. analyzed the expression profile of glucose transporters in tumor-infiltrating T cells and reported that Glut3 was more highly expressed in Treg cells than in conventional T cells. Interestingly, Treg cells exhibit low Glut3 expression in the steady state, which is in line with previous investigations [9, 11]. Accordingly, conditional deletion of Glut3 expression in Treg cells did not alter immune homeostasis and did not lead to Treg dysfunction or autoimmunity. In contrast, mice lacking Glut3 in Treg cells had better antitumor responses in a melanoma model. In addition to the upregulation of glycolysis, Glut3 expression is important for the *O*-GlcNAcylation of substrates in activated Treg cells, and this PTM is important for tumor growth, as shown by genetic *Glut3* deletion and pharmacologic inhibition of GlcNAcylation. Hence, Glut3 expression and *O*-GlcNAcylation appear to be important for the suppressive function of Tregs in the TME. Notably, Treg function was not altered in models of neuroinflammation or colitis, indicating that Glut3 targeting specifically affects TIL-Treg cells and does not lead to a general defect in immune regulation. Mechanistically, the NF-κB subunit c-Rel, which is known to be important for the function of TIL-Treg cells [13], is *O*-GlcNAcylated, enhancing the tumor-promoting function of Treg cells. Accordingly, the deletion of *Glut3* alters the NF-κB-dependent gene expression profile in TIL-Treg cells.

Notably, these findings are not specific to mice but translate to humans. The expression of *GLUT3* in three different human tumor entities correlated with the level of tumor-infiltrating Treg cells. Moreover, *GLUT3* but not *GLUT1* was more highly expressed in tumor-infiltrating Treg cells than in conventional tumor-infiltrating CD4 + T cells. The survival of patients with stomach or colon cancer was reduced in those with high *GLUT3* expression, particularly in a subgroup of patients with a high frequency of Treg cells inside the tumor. No correlation between survival and *GLUT1* expression was observed. Furthermore, the expression of *OGT* and *OGA* correlated with *GLUT3* in human tumors, and *O*-GlcNAcylation of c-Rel was detected in tumor-infiltrating Treg cells in colon cancer samples, suggesting that the Glut3*/O*-GlcNAc/c-Rel axis is evolutionarily conserved between mice and men.

**Fig. 1:**
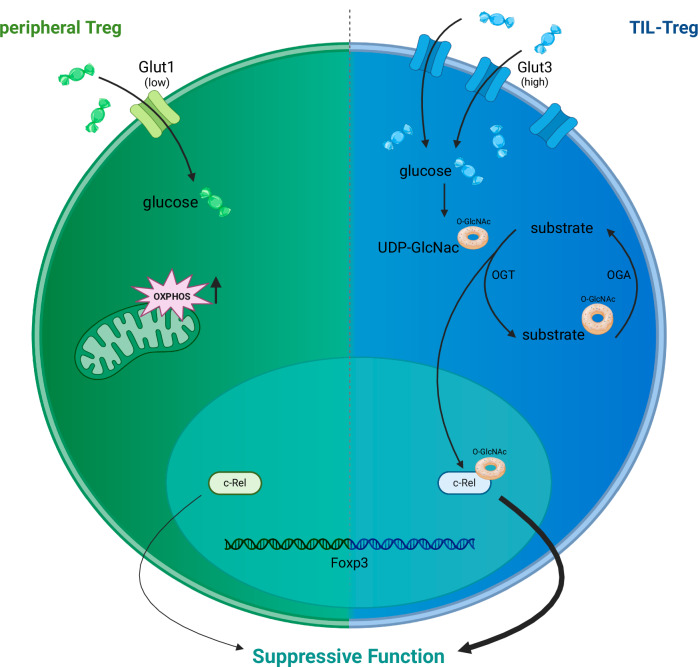
Glucose metabolism and Treg function. Peripheral Treg cells (left side) are characterized by Foxp3 expression in the nucleus, express low levels of the glucose transporter Glut1 and metabolically depend primarily on mitochondrial oxidative phosphorylation (OXPHOS). The NF-κB subunit c-Rel was shown to be important for Treg function. Tumor-infiltrating (TIL)-Treg cells (right) express high levels of Glut3 and import glucose, which is used for energy production and *O*-GlcNAcylation. Modification of substrates is mediated by *O*-GlcNAc transferase (OGT) and UDP-GlcNAc. Removal of the *O*-GlcNAc moiety is mediated by *O*-GlcNAcase (OGA). One substrate that is *O*-GlcNAcylated is c-Rel, and this modification enhances the suppressive activity of Treg cells

In summary, the study by Sharma et al. demonstrated that in tumor-infiltrating Treg cells, the glucose transporter Glut3 induces enhanced *O*-GlcNAcylation of substrates, one of which is the NF-κB subunit c-Rel (Fig. 1). *O*-GlcNAcylation of c-Rel enhances the suppressive activity of Treg cells and consequently impairs tumor immunity. As Glut3 is also connected to Th17 cells and autoimmunity [11], Glut3 and the metabolic pathways it induces are interesting therapeutic targets. However, the inhibition of Glut3 is likely achieved in a T-cell subset-specific manner since the overexpression of Glut3 in CD8 + T cells enhances tumor control [12]. It remains to be determined whether other *O*-GlcNAcylation substrates exist that regulate Treg function and whether these substrates could also be targeted. Clearly, more research on Glut3 and the metabolic changes it induces is needed. Nevertheless, the study by Sharma et al. described an important molecular mechanism by which TIL-Treg cells use a metabolic pathway to enhance their suppressive function to benefit tumors.
