# Author Correction: A special RELationship between sugar and tumor-infiltrating regulatory T cells

**DOI:** 10.1038/s41423-025-01256-z

**Published:** 2025-01-16

**Authors:** Ingo Schmitz

**Affiliations:** Department of Molecular Immunology, Ruhr University Bochum, Bochum, Germany

**Keywords:** NF-kappaB, Regulatory T cells

Correction to: *Cellular & Molecular Immunology* 10.1038/s41423-024-01248-5, published online 17 December 2024

In this article Reference 1 was incorrect and should have been ‘Sharma A, Sharma G, Gao Z, Li K, Li M, Wu M, et al. Glut3 promotes cellular O-GlcNAcylation as a distinctive tumor-supportive feature in Treg cells. Cell Mol Immunol. 2024;21(12):1474-90’. The original article has been corrected.

